# ZIF-9-Derived Cobalt and Nitrogen-Doped Carbon Nanocomposites for Sensitive Electrochemical Nitrite Determination

**DOI:** 10.3390/molecules31050768

**Published:** 2026-02-25

**Authors:** Yuan Li, Shaoqian Jia, Yuxin Shi, Lingxin Kong, Yichun Su, Guangxun Zhang, Bingyi Yan, Huan Pang, Feng Yu

**Affiliations:** 1 Key Laboratory for Green Processing of Chemical Engineering of Xinjiang Bingtuan, School of Chemistry and Chemical Engineering, Shihezi University, Shihezi 832003, China; 2 Food and Pharmaceutical Research Institute, Jiangsu Food & Pharmaceutical Science College, Huai’an 223300, China; 3 School of Materials Science and Engineering, Suzhou University of Technology, Changshu 215500, China; 4 School of Chemistry and Chemical Engineering, Yangzhou University, Yangzhou 225009, China; 5 Department of Physics, Sungkyunkwan University, 2066 Seobu-ro, Jangan-gu, Suwon 16419, Gyeonggi-do, Republic of Korea; 6 School of Petrochemical Engineering, Changzhou University, Changzhou 213164, China

**Keywords:** metal–organic frameworks, nitrite detection, electrochemical sensor, nanoparticles

## Abstract

The accurate monitoring of nitrite levels is critically important for safeguarding public health and ensuring food safety, as excessive intake presents severe risks. In this study, we developed a highly sensitive electrochemical sensor for nitrite detection utilizing a cobalt-embedded porous carbon material derived from zeolitic imidazolate frameworks (ZIFs) of ZIF-9. The precursor was subjected to pyrolysis at various temperatures, revealing that the sample carbonized at 800 °C (ZIF-9-800) exhibited superior electrocatalytic performance. This enhancement is attributed to its optimized graphitization degree, high specific surface area, and the well-dispersed active sites resulting from the in situ generated cobalt nanoparticles. The ZIF-9-800-based sensor demonstrated outstanding electrochemical performance, achieving a broad linear detection range of 0.2–7000 μM, high sensitivity (848.6 μA mM^−1^ cm^−2^), and an impressively low detection limit of 50 nM. Furthermore, the sensor exhibited excellent selectivity in the presence of common interfering ions and remarkable long-term stability, maintaining more than 80% of its initial response after extended storage. This work underscores the effectiveness of MOF-derived carbon-based catalysts, tailored through calcination temperature optimization, for constructing advanced electrochemical sensing platforms.

## 1. Introduction

ZIFs, a subclass of metal–organic frameworks (MOFs), ingeniously combine the dual advantages of traditional zeolites and MOFs, garnering extensive research interest. Notably, ZIFs exhibit exceptional chemical and thermal stability [1,2,3]. ZIF materials, such as ZIF-8, display thermal decomposition temperatures exceeding 500 °C, significantly higher than those of most conventional MOFs [4,5,6]. Moreover, ZIFs demonstrate remarkable stability in organic solvents, water, and even alkaline environments, which cannot commonly be found in many MOFs, thereby greatly broadening their practical applicability. Furthermore, numerous ZIFs can be synthesized rapidly under mild conditions (room temperature and aqueous phase) with high yield, facilitating their large-scale production and real-world application [7,8]. As a member of the ZIF family, ZIF-9 is constructed from cobalt ions (Co^2+^) and benzimidazole ligands, adopting a topology identical to that of sodalite (SOD) zeolite. In addition to the general advantages of ZIFs, ZIF-9 possesses unique features owing to its specific metal and ligand composition. The cobalt centers endow ZIF-9 with outstanding performance in oxidation catalysis, serving as active sites for various oxidative reactions [9,10,11,12]. Meanwhile, its SOD topology features uniform micropores that facilitate mass transfer of reactants and enable selective catalysis.

Nitrite (NO_2_^−^), as a key intermediate in the nitrogen cycle, is of critical importance in food safety, environmental monitoring, and public health. It is widely used as a preservative and color-fixing agent in the food industry, especially in meat processing, where it effectively inhibits the growth of pathogenic microorganisms such as Clostridium botulinum [13,14,15]. However, excessive use of nitrite can lead to high residual levels in food, posing serious threats to human health. Under acidic conditions (human stomach), nitrite can react with amines to form N-nitroso compounds (nitrosamines), which are strongly associated with an increased risk of digestive tract cancers such as esophageal and gastric cancer [16,17]. In the context of environmental monitoring, nitrite serves as an important indicator of eutrophication. Elevated nitrite levels not only damage aquatic ecosystems but also signal organic pollution in water bodies. The nitrite concentration in drinking water is strictly regulated, as excessive intake can cause methemoglobinemia—particularly in infants—leading to tissue hypoxia [18,19,20]. Conventional methods for nitrite detection include chromatography, spectroscopic techniques, and electrochemical approaches. While these methods have advantages, they often require large instruments, complex sample pretreatment, and trained operators, making them unsuitable for on-site, real-time, and rapid monitoring. Therefore, the development of novel sensing technologies that are rapid, sensitive, specific, portable, and low-cost has become a major research focus and challenge [21,22,23].

MOFs exhibit multiple advantages for nitrite detection, making them an ideal platform for constructing a new generation of high-performance sensors. The ultrahigh specific surface area of MOFs significantly enhances the enrichment capacity of nitrite molecules at the sensing interface, thereby markedly lowering the detection limit. Furthermore, complex real-world samples (such as food and blood) often contain various interfering species, such as common anions (Cl^−^, NO_3_^−^, and CO_3_^2−^), organic acids, and proteins [24,25,26,27]. The tunable pore size and surface chemistry of MOFs enable effective exclusion of these interferents through molecular sieving effects and specific interactions. Meanwhile, certain MOF structures are capable of simultaneous detection of multiple analytes, allowing for multiplex ion monitoring [28,29,30]. Despite the great promise of MOFs in nitrite sensing, several challenges remain to be addressed. For instance, the long-term stability and batch-to-batch reproducibility of MOFs under complex conditions still require improvement. In addition, the relatively high synthesis cost of some MOFs hinders their large-scale application. The development of simple and low-cost synthesis methods, such as room-temperature and aqueous-phase synthesis, is crucial to facilitate their practical deployment [31,32].

In this study, metal–carbon composites were synthesized via high-temperature carbonization of MOFs. Owing to the inherited cubic architecture and homogeneous elemental dispersion of the precursor MOFs, the resulting composites possess a well-ordered morphology and an extensive conductive network, which collectively facilitate rapid charge transfer and enhanced electrocatalytic performance. This synthetic approach effectively prevents structural disorder and excessive particle agglomeration commonly associated with conventional methods. By systematically evaluating the electrochemical behavior of samples carbonized at different temperatures, 800 °C was identified as optimal. The resulting composite exhibits exceptional sensitivity and selectivity for nitrite detection.

## 2. Results and Discussion

**Figure 1:**
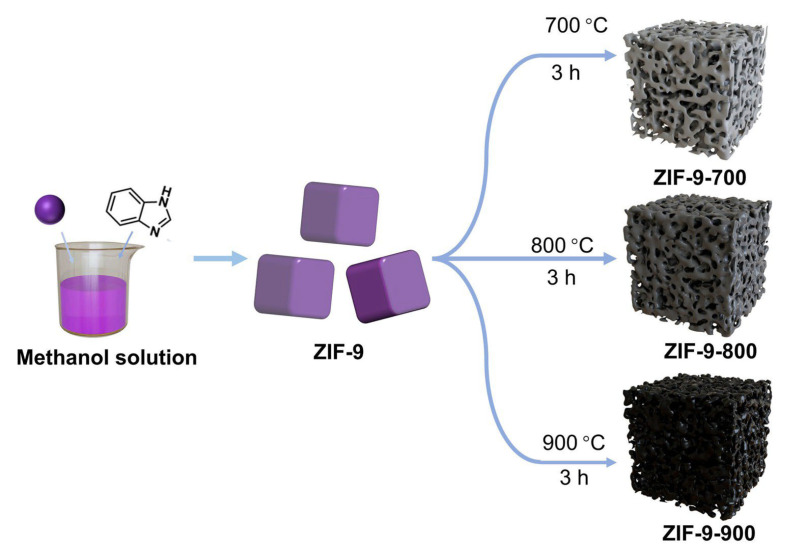
The schematic synthesis of the as-prepared ZIF-9-X cubes.

In this experiment, the ZIF-9 precursor was initially synthesized by stirring a mixture of cobalt(II) nitrate hexahydrate and benzimidazole in methanol. From the SEM, TEM, and EDX mapping images shown in Supplementary Figures S1 and S2, the ZIF-9 precursor possesses a microcube structure and the elements are distributed evenly. Given that ZIF-9 is a nitrogen-rich material, subsequent pyrolysis under a N_2_ atmosphere yielded Co/C composites. Based on thermogravimetric analysis (TGA) results (Figure S3), significant weight loss occurred within the temperature range of 700–900 °C, primarily due to thermal decomposition and evaporation of the organic ligands. Accordingly, the as-prepared ZIF-9 was subjected to thermal treatment at 700 °C, 800 °C, and 900 °C under N_2_, with the resulting samples denoted as ZIF-9-700, ZIF-9-800, and ZIF-9-900, respectively (Figure 1). Through this series of processing steps, ZIF-9-derived materials were successfully prepared at various pyrolysis temperatures, laying a foundation for further experimental evaluation.

**Figure 2:**
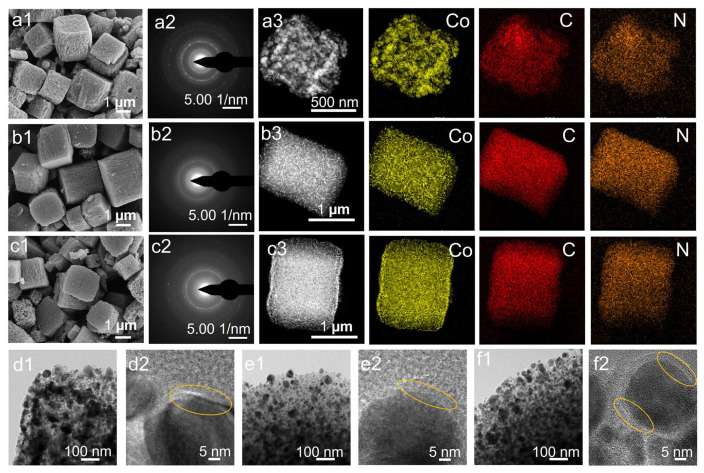
SEM images of (**a1**) ZIF-9-700, (**b1**) ZIF-9-800, and (**c1**) ZIF-9-900. SAED images of (**a2**) ZIF-9-700, (**b2**) ZIF-9-800, and (**c2**) ZIF-9-900. EDX elemental mapping (for Co, C, and N) of (**a3**) ZIF-9-700, (**b3**) ZIF-9-800, and (**c3**) ZIF-9-900. HR-TEM images of (**d1**,**d2**) ZIF-9-700, (**e1**,**e2**) ZIF-9-800, and (**f1**,**f2**) ZIF-9-900.

The morphology and particle size of ZIF-9 and its derived Co/C composites were characterized using SEM and TEM. The as-synthesized ZIF-9 particles exhibit a uniform quasi-microcube shape, with square tops measuring approximately 4–6 μm in side length and rectangular edges around 8–10 μm. These observations indicate consistent size and morphology across the ZIF-9 particles. It can be inferred that each cobalt ion in the Co(PhIM)_2_ framework is coordinated by four nitrogen atoms from the H-PhIM linkers, forming tetrahedral CoN_4_ units that further assemble into the ZIF-9 structure. The SEM images of ZIF-9-X are shown in Figure 2a1–c1. This specific configuration contributes to the material’s stability and superior properties. Furthermore, the pyrolyzed products retained a morphology similar to that of the ZIF-9 precursor, although anisotropic thermal contraction occurred with increasing carbonization temperature, leading to slightly reduced particle dimensions and the formation of rougher, more wrinkled surfaces. These results confirm the successful conversion of ZIF-9 into quasi-microcubic carbon composites while largely preserving the original morphological features, thereby incorporating characteristic carbon functionalities.

TEM analysis provided further morphological details for the ZIF-9 precursor and ZIF-9-X (Figure 2d1–f1). Well-dispersed nanoparticles were observed on the surface of ZIF-9-800 after controlled pyrolysis. The HR-TEM image of ZIF-9-X (Figure 2d2–f2) revealed lattice fringes with an interplanar spacing of 0.340 nm, corresponding to the d002 plane of graphitic carbon, indicating partial graphitization and the formation of graphitic pore walls. Additionally, lattice spacings of 0.204 nm and 0.170 nm match the d_100_ and d_200_ planes of metallic cobalt (JCPDS No. 15-0806), confirming the presence of Co nanoparticles. These unique structural and surface features facilitate the exposure of active sites and enhance electrical conductivity. Selected-area electron diffraction (SAED) as shown in Figure 2a2–c2 further verified the polycrystalline nature of ZIF-9-x. The EDX mapping imaging (Figure 2a3–c3) revealed that ZIF-9-800 consists of four elements (C, N, Co, and O), which are uniformly distributed throughout the carbon nanoparticles without detectable impurities. Notably, nitrogen doping is known to improve capacitive performance and enhance the wettability of carbon materials, thereby promoting electrolyte penetration and underpinning the application of ZIF-9-800 as an electrode material. These findings provide critical insights into the structural attributes of ZIF-9-800 and its potential applications in electronic materials.

**Figure 3:**
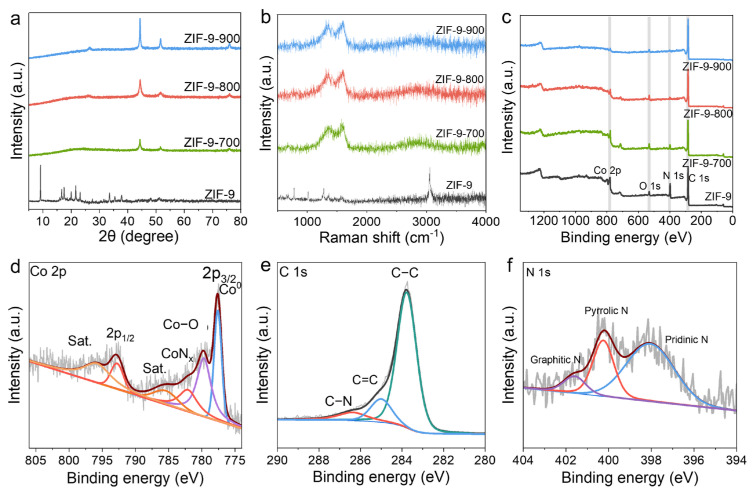
The structural characterization of the as-prepared materials. (**a**) XRD patterns of the ZIF-9 and ZIF-9-X samples. (**b**) Raman spectra of the ZIF-9-800. (**c**) XPS full spectrum of ZIF-9-800. (**d**) Co 2p XPS spectra of ZIF-9-800. (**e**) C 1s XPS spectra of ZIF-9-800. (**f**) N 1s XPS spectra of ZIF-9-800.

The structural characterization of the prepared sample is shown in Figure 3. XRD analysis was performed to investigate the composition and crystalline phases of the as-prepared samples. As shown in Figure 3a, a broad diffraction peak located at around 25° is observed for ZIF-9-X, which can be attributed to the (002) plane of graphitic carbon, confirming the presence of a graphitic phase in these samples. After calcination, the characteristic diffraction peak around 10° of the original ZIF-9 was significantly missing (Figure S4 and Figure 3a). Notably, ZIF-9-800 exhibits a higher intensity of the (002) peak compared to ZIF-9-700, indicative of a greater degree of order within the carbon structure. In addition, diffraction peaks positioned at approximately 44° and 55° correspond to the (111) and (200) planes of metallic cobalt (JCPDS No. 15-0806), further confirming the existence of Co nanoparticles in ZIF-9-X. This conclusion is also supported by HR-TEM imaging. The presence of Co nanoparticles on the surface of ZIF-9-X may contribute to enhanced electrical conductivity in these materials.

Raman spectroscopy serves as a vital tool for probing the local disorder in carbon-based materials. Analysis of Raman spectra provides critical insights into the disordered structure and key characteristics of ZIF-9 and pyrolyzed derivatives. Figure 3b displays the Raman spectra of ZIF-9 and the ZIF-9-X samples, showing two characteristic bands: the D band (1344 cm^−1^), associated with disordered carbon in the graphite lattice, and the G band (1595 cm^−1^), originating from the in-plane vibrational modes of C-C bonds in graphitic structures. The degree of structural disorder and defect density in carbon materials is commonly correlated with the intensity ratio of the D band to the G band. The calculated values for ZIF-9-700, ZIF-9-800, and ZIF-9-900 are 0.70, 0.76, and 0.81, respectively, indicating that higher pyrolysis temperatures lead to a gradual increase in defect concentration and structural disorder. This trend suggests that the sample possesses a higher defect density than the ZIF-9 precursor. An increased number of defects can effectively enlarge the specific surface area of the material, thereby enhancing its charge accumulation capability. This effect facilitates charge transfer during adsorption processes and ultimately improves the electrochemical capacitive performance of the materials.

XPS analysis techniques are used to determine the chemical states and elemental composition of ZIF-9 precursor and derivative ZIF-9-X samples’ surface by measuring the binding energy of electrons (Figure 3c). Survey scans confirmed the presence of C, N, O, and Co in all samples, consistent with previous elemental analysis results, and all originating from the ZIF-9 precursor. With increasing pyrolysis temperature, a noticeable rise in carbon content was observed, which often correlates with enhanced electrical conductivity, suggesting improved conductive properties of the materials.

Taking ZIF-9-800 as an example, deconvolution of the C 1s spectra for the ZIF-9-800 sample (Figure 3e) revealed characteristic peaks corresponding to C=N, C=C, and C-C bonds. The presence of C=N signals suggests possible nitrogen doping within the carbon framework. Furthermore, the N 1s spectra (Figure 3f) displayed three fitted peaks, assigned to pyridinic N, pyrrolic N, and graphitic N, indicating that nitrogen atoms were effectively incorporated and stabilized in the carbon matrix. Such doping is known to improve both electrical conductivity and hydrophilicity. Although the relative nitrogen content decreased due to the thermal decomposition of C=N bonds at elevated temperatures, it remained considerably high compared to values reported in other studies. Analysis of the Co 2p spectra (Figure 3d) for the ZIF-9-800 samples showed characteristic peaks corresponding to metallic Co (Co^0^), Co-O, Co-Nx, and satellite features. The presence of metallic Co is consistent with XRD results, confirming the formation of Co nanoparticles. The Co-O peak likely originates from surface oxidation of cobalt, while the Co-N_x_ species may be derived from residual Co-N_4_ moieties inherited from the ZIF-9 structure, in agreement with FT-IR analysis. The persistence of N_x_ coordination across all three samples further suggests that the framework integrity was largely maintained despite increasing pyrolysis temperatures. According to the XPS spectra shown in Figures S5–S7 and the elemental content analysis in Supplementary Table S1, the results show that the carbon content will increase with the increase in calcination temperature, which means an improvement in conductivity and stability.

To evaluate the specific surface area and pore size distribution of the obtained samples, N_2_ adsorption–desorption curves were collected for both ZIF-9 and the ZIF-9-X derivatives. As shown in the corresponding Supplementary Figures S8–S11, ZIF-9 exhibits a Type III isotherm, characterized by negligible N_2_ uptake at low relative pressure (P/P_0_ < 0.1) and a sharp adsorption increase at high relative pressure (0.6 < P/P_0_ < 1.0), indicating the lack of a well-developed porous structure. In contrast, the ZIF-9-X materials display composite Type IV and V isotherms. The rapid uptake at low pressure suggests the presence of micropores, while the distinct hysteresis loop observed in the range of P/P_0_ = 0.5–1.0 is indicative of mesoporosity. In particular, the ZIF-9-800 sample shows a pronounced hysteresis loop, implying abundant porosity and large pore volume. In fact, ZIF-9 shows significant mesoporous characteristics. In contrast, the ZIF-9-X materials, especially ZIF-9-800, exhibit a broader pore size distribution. This indicates the development of a more complex and disordered porous architecture with mixed micro- and mesoporosity, which is a common outcome of high-temperature carbonization. Despite the increased disorder, this heterogeneous pore network contributed to a large overall pore volume, which is beneficial for electrolyte infiltration and mass transport during the electrocatalytic process. The results clearly demonstrate the development of a well-defined porous architecture in the ZIF-9-X samples obtained at higher carbonization temperature.

**Figure 4:**
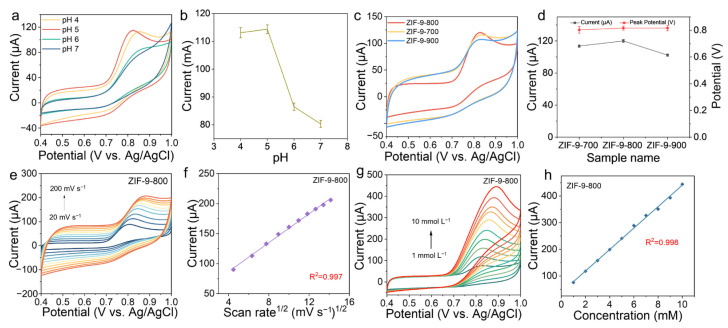
(**a**) CV profiles of ZIF-9-800 at different pH in 0.1 M PBS (Phosphate-Buffered Saline) electrolyte containing 3 mM nitrite at 50 mV^−1^. (**b**) The current of the ZIF-9-800 electrode at different pH. (**c**) CV profiles of different electrodes and (**d**) corresponding current. (**e**) CV profiles of ZIF-9-800 electrode in 0.1 M PBS with 3 mM nitrite between 20 and 200 mV s^−1^. (**f**) The plot of the ZIF-9-800 sample showing the peak current vs. the square root of the scan rate. (**g**) CV curves of the ZIF-9-800 sample measured in 0.1 M PBS (pH 5.0) with increased nitrite concentrations of 1–10 mM. (**h**) Calibration profiles of ZIF-9-800 showing the peak current vs. the nitrite concentration.

The electrochemical responses of ZIF-9-derived Co/C composites calcined at different temperatures were compared using cyclic voltammetry (CV) in Phosphate-Buffered Saline (PBS) containing 3 mM nitrite. Since the pH of real-world samples often varies and can be complex, understanding the sensor’s performance across different pH levels helps determine its effective operating range and minimize interference from other substances. Figure 4a,b reveal that pH significantly influences both the speciation and electrochemical oxidation behavior of nitrite. A pH of 5.0 was identified as the optimal condition for detection, likely because nitrite exists in a mixture of HNO_2_ and NO_2_^−^ at this pH, which most favorably promotes the oxidation kinetics at the electrode surface. Under strongly acidic conditions, NO_2_^−^ reacts with excess H^+^ to form HNO_2_. However, nitrous acid is unstable and readily decomposes to produce NO gas, which escapes from the solution. This causes a loss of nitrite from the sample, leading to low measurement results [33,34]. Figure 4c,d indicate that all samples exhibited a distinct and sharp anodic oxidation peak around 0.8 V (vs. Ag/AgCl), with the ZIF-9-800 sample demonstrating a higher peak current (I_p_) than the others. This suggests that selecting an appropriate calcination temperature is an effective strategy for enhancing the electrochemical sensing performance of ZIF-9-derived precursors. The pronounced electrocatalytic activity of ZIF-9-800 for nitrite oxidation can be fundamentally attributed to its enhanced electrical conductivity, facilitated by the graphitic carbon matrix, and the presence of highly active sites provided by the well-dispersed cobalt nanoparticles. This synergistic effect between the conductive support and the active sites significantly lowers the overpotential and boosts the current response for the reaction.

The influence of different scan rates (from 20 mV s^−1^ to 200 mV s^−1^) on the oxidation peak behavior of the ZIF-9-800 sample was investigated at a fixed nitrite concentration of 3 mM. As shown in Figure 4e, with increasing scan rate, the oxidation peak current gradually increased, while the oxidation peak potential shifted noticeably in the positive direction. This phenomenon is primarily attributed to an irreversible electrode process. The positive shift of E_p_ with scan rate indicates that the oxidation reaction involves a substantial energy barrier, where the electron transfer rate is relatively slow and constitutes the rate-determining step. This provides key insight for determining the type of reaction. By linearly fitting the peak current against the square root of the scan rate (v^1/2^), a highly linear proportional relationship between I_p_ and v^1/2^ was observed, with a linear correlation coefficient (R^2^) of 0.997 (Figure 4f). This result conclusively demonstrates that the rate of the nitrite oxidation reaction on ZIF-9-800 is predominantly controlled by the mass transport process—specifically, the diffusion of nitrite molecules/ions from the bulk solution to the electrode surface—rather than by the kinetics of the surface chemical reaction.

Additionally, CV responses were tested under varying nitrite concentrations (from 1 mM to 10 mM). Figure 4g shows that as the nitrite concentration increased progressively, the anodic oxidation peak current exhibited a continuous and regular enhancement, while the oxidation peak potential remained largely unchanged. The increase in current with concentration is a fundamental and essential characteristic enabling quantitative detection of the analyte, indicating a strong correlation between the electrocatalytic reaction at the electrode surface and the analyte concentration. The stability of the oxidation peak potential suggests that the catalytic mechanism remains consistent and stable across the concentration range tested, with no signs of electrode poisoning or passivation, thereby ensuring reproducible and reliable detection. Based on these data, the oxidation peak current values corresponding to different nitrite concentrations were extracted and linearly fitted against concentration to construct a calibration curve. As illustrated, within the broad concentration range of 1–10 mM, an excellent linear relationship between peak current and nitrite concentration was achieved, with a linear correlation R^2^ as high as 0.998 (Figure 4h). The width of the linear range and the quality of the linear correlation directly indicate the reliability and applicability of the sensor in practical applications. We further conducted electrochemical impedance spectroscopy (EIS) measurements on ZIF-9, ZIF-9-700, ZIF-9-800, and ZIF-9-900 samples after the reaction. As shown in Figure S12, the Nyquist plots of the electrodes all exhibited a raised semicircle in the high-frequency region, representing the charge transfer resistance (Rct) within the active material, and a straight line in the low-frequency region, representing the ionic diffusion behavior within the active material. The ZIF-9-800 electrode exhibited a smaller charge transfer resistance and superior ionic diffusion behavior, indicating that it possesses excellent ionic and electronic conductivity. Therefore, the ZIF-9-800 electrode can provide sufficient electrons to the catalytically active sites, thereby lowering the overpotential of the nitrite oxidation reaction. Furthermore, the excellent conductivity of the ZIF-9-800 electrode ensures stable and consistent electron transport within and across the electrode surface, avoiding current signal deviations caused by fluctuations in electron transfer.

Real-world samples, such as food extracts, environmental water, and bodily fluids, exhibit highly complex chemical compositions. Numerous electroactive substances (inorganic ions, organic acids, sugars, and amino acids, among others) may undergo oxidation or reduction reactions at the working potential of the sensor, thereby generating interfering signals [35,36]. By introducing common interfering species at high concentrations (typically several multiples of the target analyte concentration) and testing whether they induce significant current responses at the nitrite detection potential, one can effectively evaluate the sensor’s performance under simulated real-world conditions. In the experiment, a small amount of nitrite (20 μM) was first added to establish a baseline signal, followed by the introduction of various common interferents at tenfold higher concentrations, including inorganic ions (Cu^2+^, Zn^2+^, Mg^2+^, Al^3+^, Na^+^, Ca^2+^, and K^+^) and organic molecules (glucose (GLU), dopamine (DA), uric acid (UA), and ascorbic acid (AA)).

**Figure 5:**
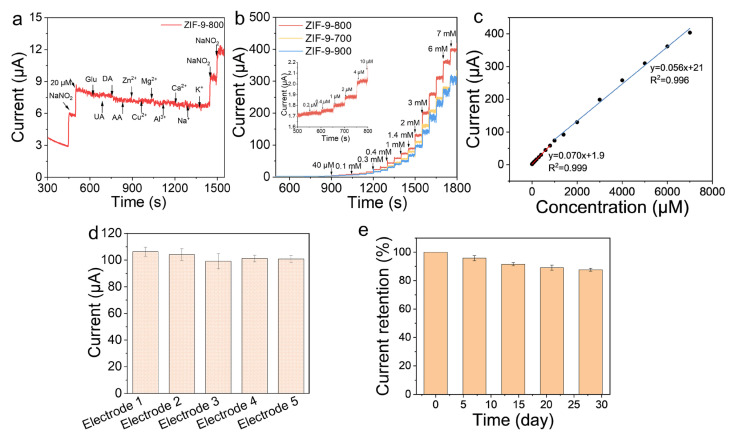
(**a**) Selectivity of ZIF-9-800. (**b**) CA curves of ZIF-9-X in 0.1 M PBS (pH 5.0) with 3 mM nitrite at 0.90 V. (**c**) Calibration curves of ZIF-9-800 showing the detection current vs. nitrite concentration. (**d**) Reproducibility of nitrite response at the ZIF-9-800 electrode. (**e**) Stability of ZIF-9-800.

Based on the CV results, the anodic peak for ZIF-9-800 is situated at approximately 0.9 V. This potential facilitates the highest oxidation rate and current output for nitrite, which is crucial for achieving high sensitivity. Thus, 0.9 V was adopted as the working potential. As shown in Figure 5a, these high levels of interferents did not provoke significant current responses. In contrast, each addition of nitrite produced a distinct and reproducible current step, visually confirming the sensor’s exceptional selectivity toward nitrite. To more intuitively demonstrate the sensor’s responsiveness to changes in concentration, the current–time response curve of the ZIF-9-800 sample was recorded during successive, equal-volume additions of nitrite standard solution. Chronoamperometry was selected as the detection method for constructing the calibration curve because it is well-established for achieving high sensitivity in quantitative analysis. This is attributed to its operation at a fixed potential, which minimizes the charging current contribution and allows for the precise measurement of the steady-state Faradaic current directly related to the analyte concentration. Figure 5b shows that after each addition of nitrite, the ZIF-9-800 material produced clearer and larger current step signals, indicating a markedly enhanced electrocatalytic response to nitrite. The corresponding calibration curve (Figure 5c) revealed a linear relationship between the current signal and nitrite concentration. Based on the concentration levels, the curve is divided into two segments (0–800 μM and 1000–8000 μM) for fitting separately. R^2^ value of 0.999 and 0.996 affirms a highly reliable linear fit, indicating that the sensor possesses excellent applicability for practical use. Based on calculations, the sensitivity of the ZIF-9-800 sensor reached 848.6 μA mM^−1^ cm^−2^. Reproducibility was investigated by fabricating five independent electrodes under identical conditions. The oxidation peak currents (Figure 5d) exhibited a minimal relative standard deviation (RSD) of around 3%, confirming exceptional repeatability in sensor fabrication and consistent operational reliability. The long-term stability of the ZIF-9-800 sensor was evaluated by storing the electrodes at 5 °C and performing periodic electrochemical measurements over 30 days (Figure 5e). After this period, the oxidation peak current retained approximately 90% of its initial value, underscoring the robust and reliable operational stability of the nitrite detection platform. The diminished current response observed in the A-based nitrite sensor is largely due to the limited availability of active sites. This phenomenon stems from the aggregation of Co nanoparticles in the ZIF-9-800 framework during extended immersion or cyclic potential sweeps, leading to a decrease in specific surface area and consequently fewer active sites. Moreover, several other interference factors exist within the measurement system. Specifically, dissolved oxygen and accumulated impurity ions in the electrolyte may occupy electrode active sites competitively with nitrite, leading to the production of interfering currents. Furthermore, shifts in the electrolyte pH outside the suitable window for nitrite oxidation can diminish the reaction kinetics.

The findings position this platform as a promising candidate for practical electrochemical sensing applications, particularly in environments demanding high precision and durability. Lastly, the practical feasibility of ZIF-9-800/GCE was evaluated by adding nitrite to sausage samples. Aliquots of 60, 70, and 90 μM of nitrite were introduced consecutively, and the obtained results were in good agreement with the expected values. Each experiment was repeated three times to verify the reproducibility of the sensor. As shown in Table 1, the recoveries for the sausage samples ranged from 104% to 109%, with relative standard deviations (RSDs) less than 2.8%. These results demonstrate that ZIF-9-800 exhibits excellent performance in nitrite detection and possesses high reliability for practical applications. Moreover, the performance of the as-prepared materials was investigated against various other sensors, and the findings can be found in Table 2. Nitrite quantification using ZIF-9-800 demonstrated a wide linear range, superb sensitivity, and a comparatively low LOD (limit of detection). In conclusion, these results highlight the outstanding selectivity, sensitivity, stability, and reproducibility of the ZIF-9-800-based sensor for nitrite detection.

## 3. Conclusions

The strategic synthesis of metal/carbon–nitrogen composites through the controlled pyrolysis of MOFs represents a successful methodology for constructing high-performance electrochemical nitrite sensors. The pristine MOF skeleton provides substantial porosity and a high specific surface area, facilitating abundant active sites for electrocatalytic reactions. Subsequent carbonization yields a robust conductive carbon matrix that enhances structural integrity and promotes efficient electron transfer within the composite material. This synergistic configuration endows the sensor with an exceptionally low detection limit of 50 nM for nitrite and remarkable selectivity against common interfering species. Electrochemical assessments of the ZIF-9-800 modified glassy carbon electrode demonstrated a wide linear response range from 0.2 to 7000 μM (R^2^ = 0.997), high sensitivity, and sustained sensing performance after prolonged storage, underscoring its operational durability. To sum up, this work highlights the significant potential of MOF-derived carbon-based architectures as advanced electrochemical sensing platforms. It provides a novel perspective on the functional application of MOF-derived nanocomposites in chemical sensing, paving the way for the design of next-generation analytical devices.

## Figures and Tables

**Table 1:** Determination of nitrite in sausage samples.

Sample	Added (μM)	Found (μM)	Recovery (%)	RSD (%, *n* = 4)
Sausage	60	65.6	109	2.63
Sausage	70	75.0	107	0.66
Sausage	90	93.3	104	2.72

**Table 2:** Comparison of sensitivity for nitrite detection by various sensors.

Electrodes	Electrolyte	Sensitivity (μA mM^−1^ cm^−2^)	Linear Range (μM)	LOD (μM)	Ref.
NiCo-RGO	0.1 M PBS (pH = 5)	67.67	60–860	18	[37]
Pt/Ni/NCNTs	0.1 M KCl	276.92	0.5–40	0.17	[38]
Dendrimer/AuNPs I/GC	0.1 M NaClO_4_ (pH = 6.5)	640	10–5000	0.2	[39]
PD-LSB/CS/GCE	0.1 M KCl (pH = 3)	275	200–30,000	1.08	[40]
CuCP-MWCNTs-SPCE	0.1 M KCl (pH = 7.4)	163.72	100–10,000	1.5	[41]
MOF-525/GNR	0.1 M KCl	96.4	100–2500	0.75	[42]
GO-CS-AuNPs/GCE	0.1 M PBS (pH = 5)	-	0.9–18.9	0.30	[43]
ION-rGO	0.1 M PBS (pH = 7)	-	0.1–10	13 pm	[44]
ZIF-9-800	0.1 M PBS (pH = 5)	848.6	0.2–7000	0.05	This work

## Data Availability

Data available upon request due to restrictions.

## Supplementary Materials

The following supporting information can be downloaded at https://www.mdpi.com/article/10.3390/molecules31050768/s1. Experimental Section; Figure S1. EDX mapping images of ZIF-9; Figure S2. (a) SEM of ZIF-9 cubes. (b) TEM images of ZIF-9 cubes; Figure S3. TGA curves of ZIF-9; Figure S4. XRD image of ZIF-9 cubes; Figure S5. XPS spectra of ZIF-9. (a) Co2p. (b) C 1s. (c) N 1s; Figure S6. XPS spectra of ZIF-9-700. (a) Co2p. (b) C 1s. (c) N 1s; Figure S7. XPS spectra of ZIF-9-900. (a) Co2p. (b) C 1s. (c) N 1s; Figure S8. (a) Nitrogen adsorption/desorption isotherm of ZIF-9. (b) Pore size distribution of ZIF-9; Figure S9. (a) Nitrogen adsorption/desorption isotherm of ZIF-9-700. (b) Pore size distribution of ZIF-9-700; Figure S10. (a) Nitrogen adsorption/desorption isotherm of ZIF-9-800. (b) Pore size distribution of ZIF-9-800; Figure S11. (a) Nitrogen adsorption/desorption isotherm of ZIF-9-900. (b) Pore size distribution of ZIF-9-900; Figure S12. The EIS plots of ZIF-9, ZIF-9-700, ZIF-9-800, and ZIF-9-900; Table S1. XPS elemental content analysis in ZIF-9 and ZIF-9-X samples.
