# Supplementary material for: ZIF-9-Derived Cobalt and Nitrogen-Doped Carbon Nanocomposites for Sensitive Electrochemical Nitrite Determination
Source: Molecules. 2026 Feb 25;31(5):768. doi: 10.3390/molecules31050768 (PMC12985869; doi:10.3390/molecules31050768)
Supplement: Supplementary file 1 [file molecules-31-00768-s001.zip › molecules-4073542-supplementary.pdf]

Supporting Information

ZIF-9-Derived Cobalt and Nitrogen-Doped Carbon Nanocomposites for sensitive Electrochemical Nitrite Detection

Yuan Li, Shaoqian Jia, Yuxin Shi[*]*,Lingxin Kong, Yichun Su, Guangxun Zhang, Bingyi Yan,*

Huan Pang[*]*, Feng Yu*[*]

Yuan Li and Feng Yu-Key Laboratory for Green Processing of Chemical Engineering of Xinjiang Bingtuan, School of Chemistry and Chemical Engineering, Shihezi University, Shihezi 832003, China

Yuan Li and Shaoqian Jia-Jiangsu Food and Pharmaceutical Science College, 4 E Meicheng Rd., Huai'an 223003, China

Yuxin Shi-School of Materials Science and Engineering, Suzhou University of Technology, Changshu 215500, China

Lingxin Kong, Guangxun Zhang, Bingyi Yan and Huan Pang-School of Chemistry and Chemical Engineering, Yangzhou University, Yangzhou 225009, China

Lingxin Kong-Department of Physics, Sungkyunkwan University, 2066, Seobu-ro, Jangan-gu, Suwon, Gyeonggi-do 16419, Republic of Korea

Yichun Su-School of Petrochemical Engineering, Changzhou University, Changzhou 213164, China

Key words: Metal-organic frameworks, Nitrite detection, Electrochemical sensor, Nanoparticles

Experimental Section

1.1 Materials and reagents

Cobalt nitrate hexahydrate ($Co(NO_3)_2·6H_2O$), benzimidazole ($C_7H_6N_2$), cetyltrimethylammonium bromide ($C_{19}H_{42}BrN$), triethylamine ($C_6H_{15}N$) and methanol (CH_3OH) were purchased from Shanghai Sinopharm Chemical Reagent, co. Ltd. All the chemicals used in this study are of analytical grade and used without purification. Ultrapure water with a resistance of 18.2 MΩ (Thermo Fisher Scientific co. Ltd, USA) was used throughout all experiments.

1.2 Preparation of ZIF-9

ZIF-9 was synthesized via a room-temperature stirring method. The detailed experimental procedure is as follows: First, benzimidazole (0.118 g) and cobalt(II) nitrate hexahydrate (0.14 g) were completely dissolved in 15 mL and 10 mL of methanol, respectively, forming Solution A and Solution B. Then, 30 μL of triethylamine was added to Solution A under stirring. Meanwhile, 5 mg of cetyltrimethylammonium bromide (CTAB) was introduced into Solution B, followed by ultrasonication for 15 minutes. Subsequently, Solution B was added dropwise to Solution A under continuous stirring at 600 rpm for 1 h at room temperature. The mixture was then allowed to stand undisturbed for 6 h, resulting in a purple suspension containing ZIF-9.

1.3 Preparation of ZIF-9-X (X=700, 800, 900)

The ZIF-9 nanoparticles were collected by centrifugation at 10000 rpm for 2 min, and the precipitate was redispersed in methanol and washed three times. The resulting purple precipitate was dried in a vacuum oven at 60 °C for 12 h to obtain solid ZIF-9 particles. The as-synthesized ZIF-9 crystals were further subjected to thermal treatment under N_2 atmosphere for 3 h at heating rates of 5 $°C·min^{-1}$. The calcination temperatures were set at 700, 800, and 900 °C, respectively.

1.4 Characterization

Scanning electron microscope (SEM, Zeiss Gemini SEM 300) and transmission electron microscope (TEM, FEI Tecnai G2 F30; FEI Co., USA) were used to characterize the morphology of the samples. Bruker D8 Advance X-ray diffractometer (XRD) (Cu Kα λ = 0.15406 nm, Bruker, Germany) and Thermo escalab 250 Xi X-ray photoelectron spectroscopy (Thermo Fisher Scientific, US) was used to characterize the phase compositions and electronic states of the samples.

1.5 Electrochemical measurements

The ZIF-9-X (X=700, 800, 900) ink was prepared by dispersing 3.0 mg catalyst in a mixed solvent containing 250 μL ethanol, 220 μL deionized water, and 30 μL 5 % Nafion solution (DuPont), followed by 30-min ultrasonication. A 5-μL aliquot of this homogeneous suspension was drop-cast onto a mirror-polished glassy carbon electrode (GCE, 3 mm diameter; geometric area: 0.0707 cm^2). The modified electrode was dried at 40 °C for 30 min before electrochemical testing.

Various electrochemical tests were performed using a conventional three-electrode setup on an Electrochemical Workstation CHI 760E (manufactured in Chen Hua, China). The ZIF-9-X modified GCE, Ag/AgCl, and the platinum wire were chosen as the working electrode, the reference electrode, and the counter electrode, respectively. The electrochemical properties of different electrodes were studied by cyclic voltammetry (CV) and in 0.1 M PBS (137 mM NaCl and 2.7 mM KCl, pH 5.0) containing 3 mM nitrite. The 0.1 M PBS (pH 5.0) was prepared by dissolving disodium hydrogen phosphate, sodium dihydrogen phosphate, sodium chloride, and potassium chloride in deionized water, and the pH was adjusted using HCl or NaOH solutions. The optimization of pH (4.0-7.0) was conducted by CV at a scan rate of 50 mV s^{-1} in 0.1 M PBS (pH=5.0) containing 3 mM nitrite. The selectivity of the developed nitrite sensor was investigated in the presence of a variety of possible interfering substances, including the examination of Na^+, glucose, dopamine, uric acid, and ascorbic acid, Ca^{2+},

K^+, Mg^{2+}, Al^{3+}, and Zn^{2+} using an amperometric technique in 0.1 M PBS. To verify the stability of the material, we stored the electrodes at a constant temperature of 5 ºC in a refrigerator for 28 days to verify the stability of the sensor material.

Sensitivity test: The sensitivity of the sensor was calculated based on the slope of the calibration curve obtained from the chronoamperometric (i-t) measurements. The specific calculation formula and steps are as follows: Plotting the Calibration Curve: The steady-state current response (ΔI, unit: μA) from the chronoamperogram was plotted against the corresponding concentration of nitrite (C, unit: mM). Linear Fitting: The data points within the linear range were fitted using the least-squares method to obtain a linear regression equation in the form of I = kC + b, where k is the slope of the curve (unit: μA mM^{-1}). Normalization by Electrode Area: To report the sensitivity as a current density (which allows for comparison between sensors of different sizes), the slope (k) was divided by the geometric surface area (A) of the glassy carbon electrode (GCE) used in our experiments (A = 0.07 cm^2). Sensitivity (S) = k/A. Therefore, the reported sensitivity of 848.6 μA mM^{-1} cm^{-2} was derived from the slope of the calibration curve (Figure 5c) divided by the electrode area.

Sausage pretreatment procedure: Firstly, 10 g sausage sample was homogenized into mash and then mixed with 25 mL of 100 g L^{-1} saturated borax solution. Then, 300 mL of 70 °C deionized (DI) water was added, and the mixture was heated at boiling point for 15 min. After cooling down the mixture to room temperature, 10 mL of potassium ferrocyanide solution (212 g L^{-1}) and 5 mL of zinc acetate solution (440 g L^{-1}) were added to precipitate the protein. Next, the mixture was diluted to 400 mL with DI water and filtered after 40 min of standing to remove the upper fat. The resulting sample extract was stored at 3-4 °C in the fridge.

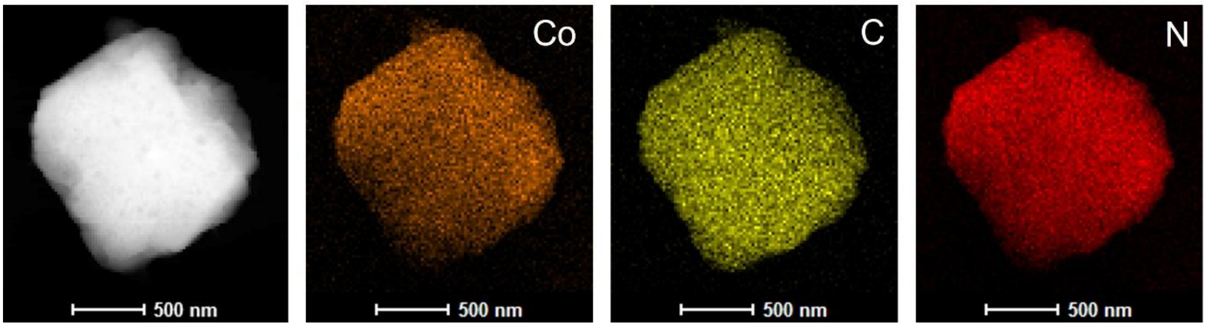

Figure S1. EDX mapping images of ZIF-9.

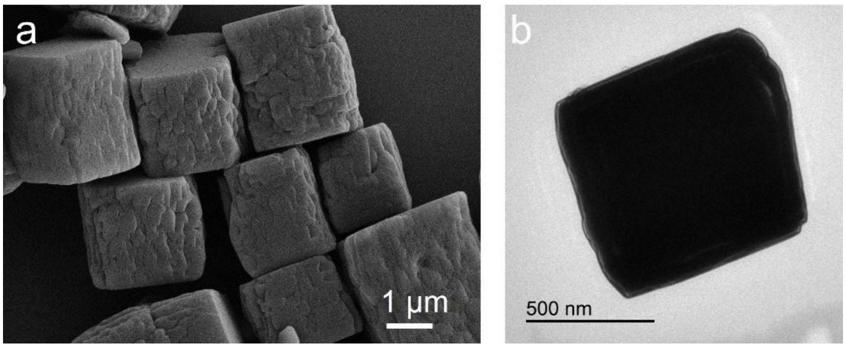

Figure S2. (a) SEM of ZIF-9 cubes. (b) TEM images of ZIF-9 cubes.

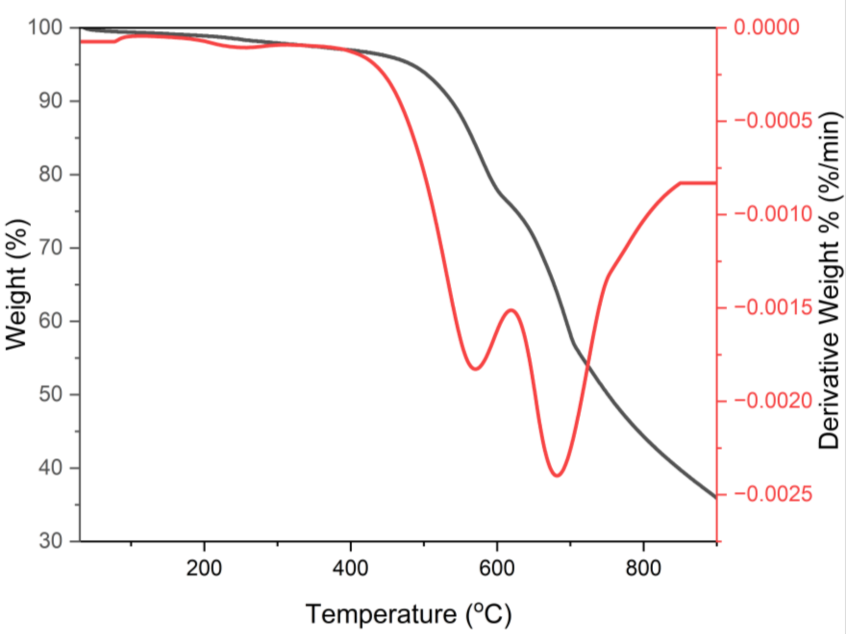

Figure S3. TGA curves of ZIF-9.

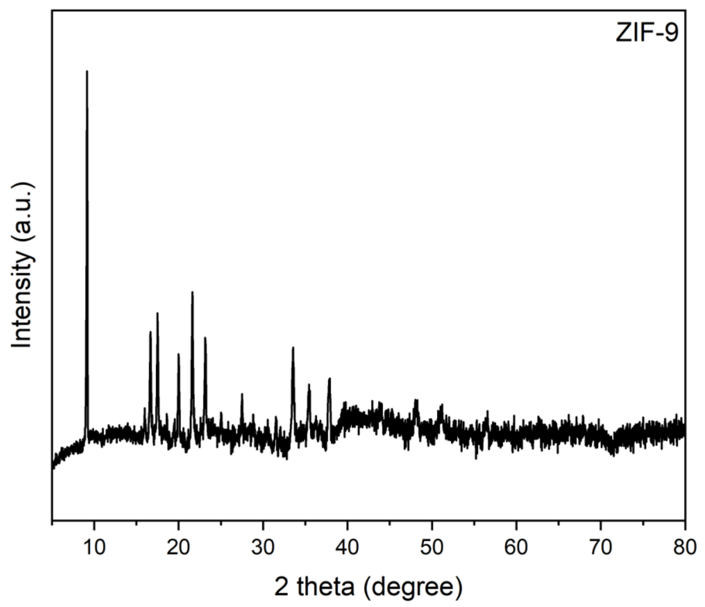

Figure S4. XRD image of ZIF-9 cubes.

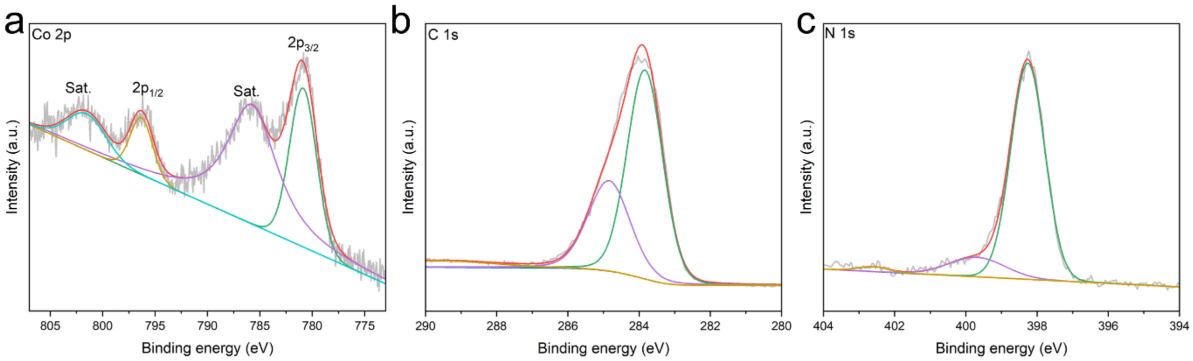

Figure S5. XPS spectra of ZIF-9. (a) Co2p. (b) C 1s. (c) N 1s.

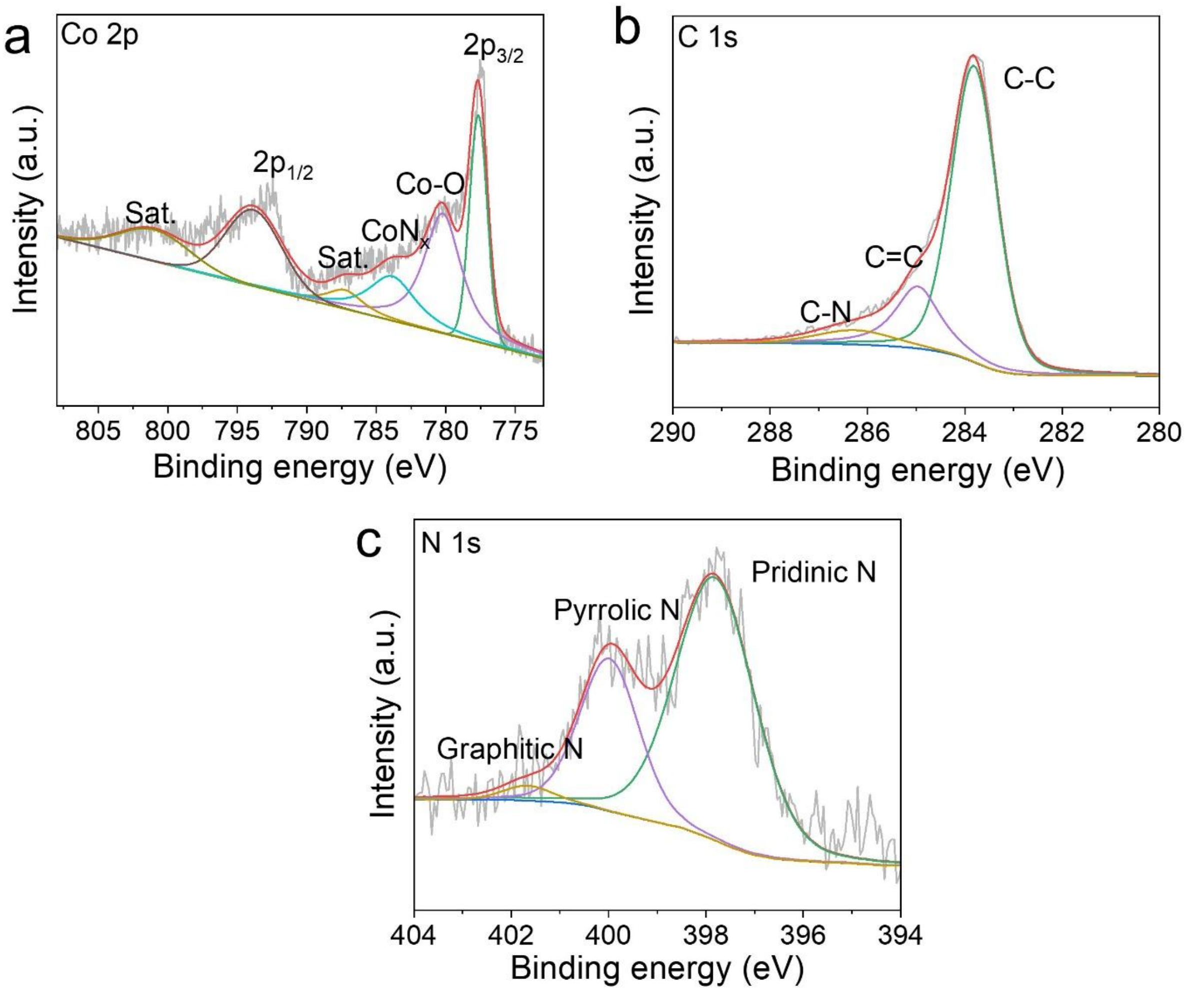

Figure S6. XPS spectra of ZIF-9-700. (a) Co2p. (b) C 1s. (c) N 1s.

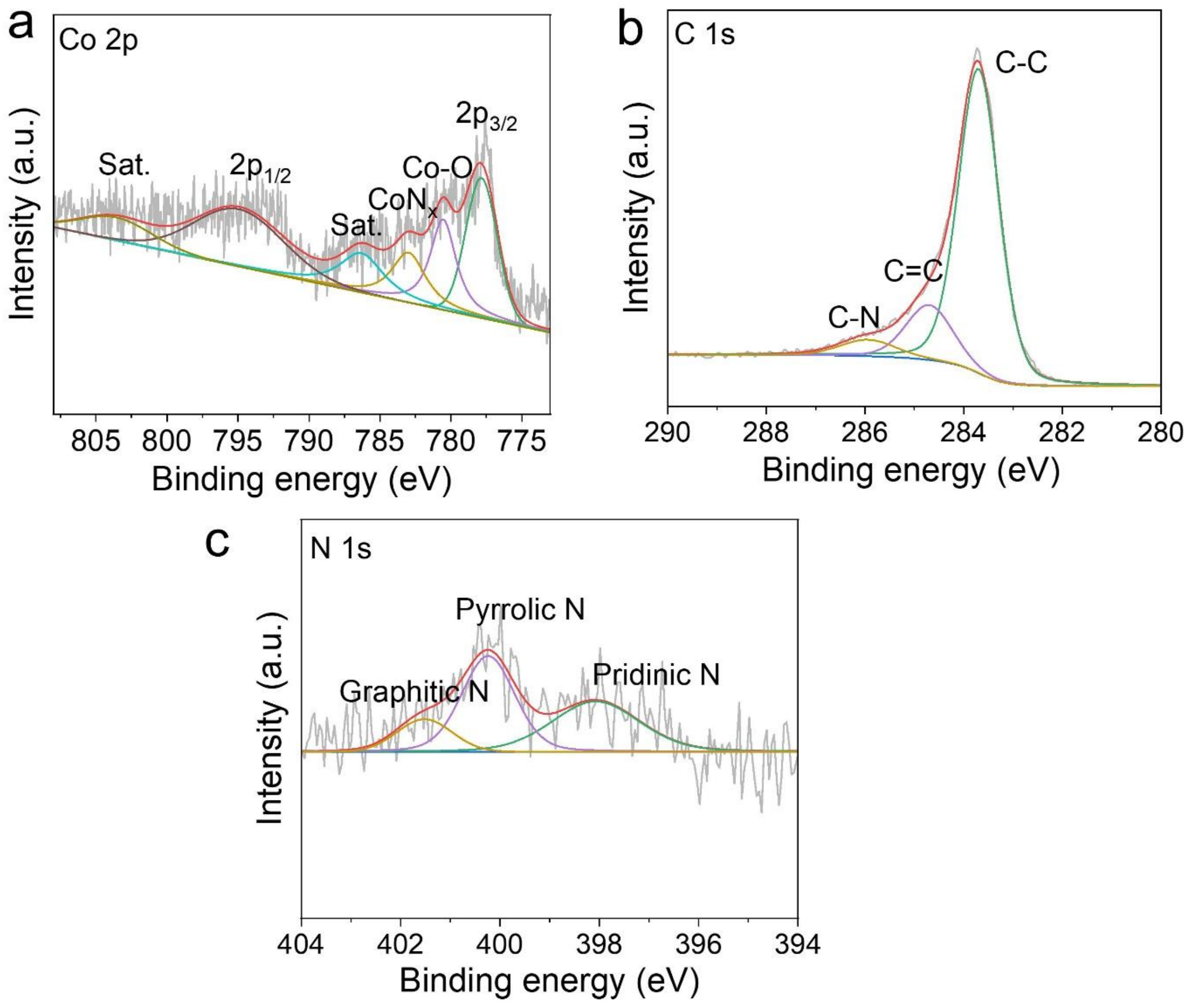

Figure S7. XPS spectra of ZIF-9-900. (a) Co2p. (b) C 1s. (c) N 1s.

Table S1. XPS elemental content analysis in ZIF-9 and ZIF-9-X samples.

Sample	Carbon/C (at.%)	Carbon/N (at.%)	Carbon/Co (at.%)	Carbon/O (at.%)
ZIF-9	70.62	20.78	5.47	3.13
ZIF-9-700	71.52	11.67	8.63	8.19
ZIF-9-800	77.57	9.12	5.68	7.63
ZIF-9-900	81.25	7.84	4.70	6.21

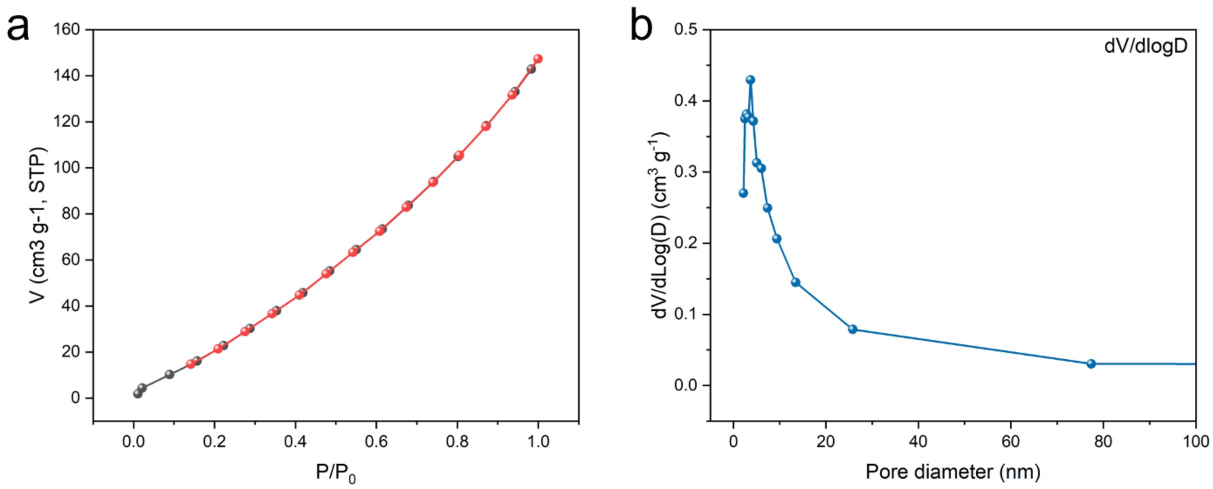

Figure S8. (a) Nitrogen adsorption/desorption isotherm of ZIF-9. (b) Pore size distribution of ZIF-9.

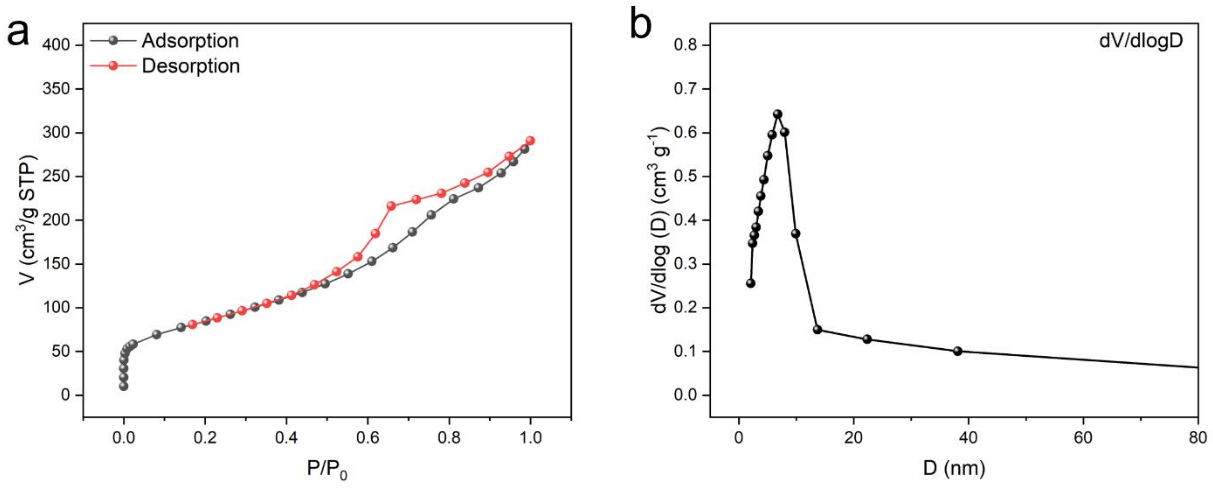

Figure S9. (a) Nitrogen adsorption/desorption isotherm of ZIF-9-700. (b) Pore size distribution of ZIF-9-700.

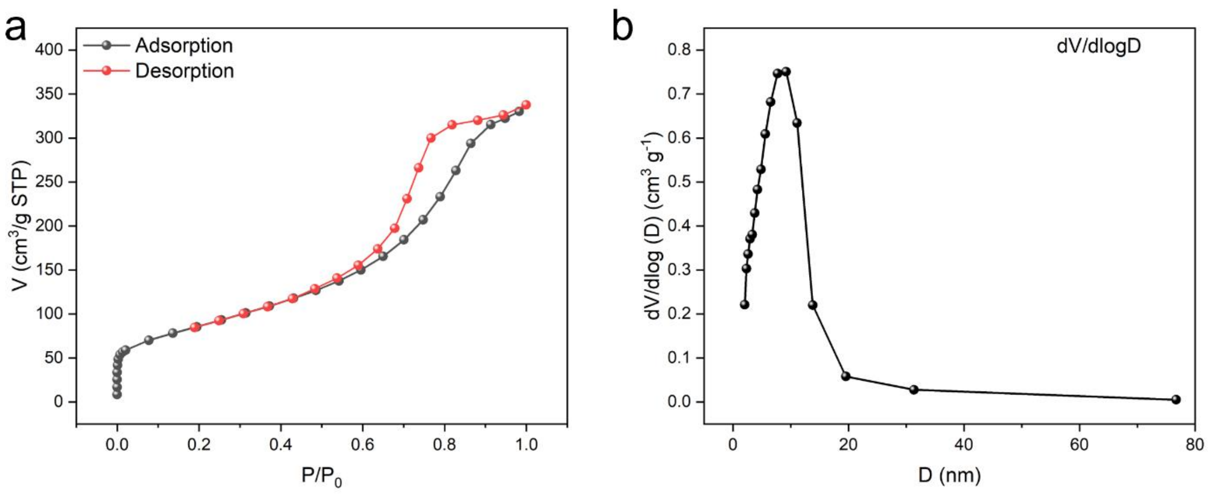

Figure S10. (a) Nitrogen adsorption/desorption isotherm of ZIF-9-800. (b) Pore size distribution of ZIF-9-800.

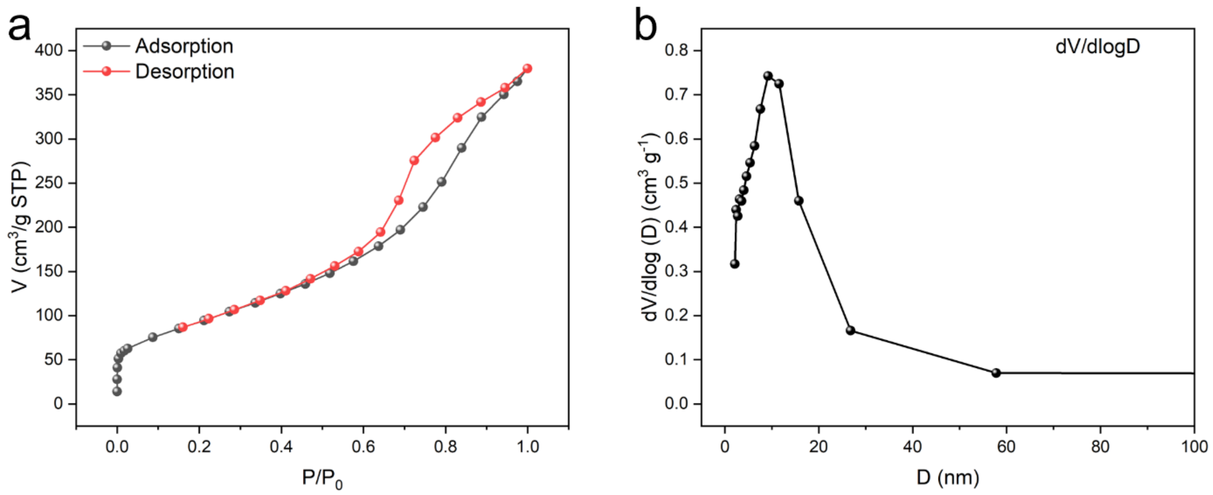

Figure S11. (a) Nitrogen adsorption/desorption isotherm of ZIF-9-900. (b) Pore size distribution of ZIF-9-900.

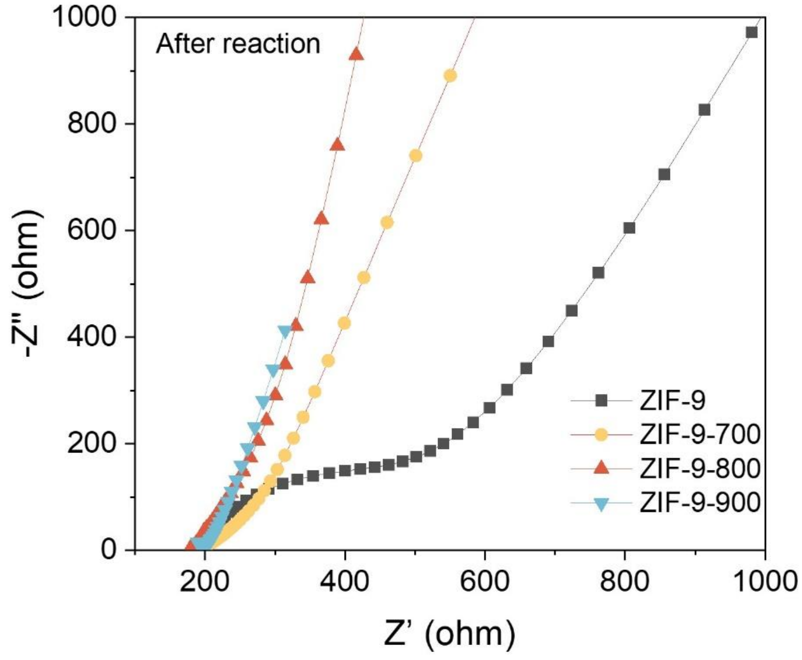

Figure S12. The EIS plots of ZIF-9, ZIF-9-700, ZIF-9-800, and ZIF-9-900.
